# Human-centered design of a novel soft exosuit for post-stroke gait rehabilitation

**DOI:** 10.1186/s12984-024-01356-3

**Published:** 2024-04-24

**Authors:** Chandramouli Krishnan, Olugbenga P. Adeeko, Edward Peter Washabaugh, Thomas E Augenstein, Maureen Brudzinski, Alyssa Portelli, Claire Zabelle Kalpakjian

**Affiliations:** 1 School of Kinesiology, University of Michigan, Ann Arbor, MI USA; 2 Elite Athlete Products, Inc, San Diego, CA USA; 3 Department of Biomedical Engineering, Wayne State University, Detroit, MI USA; 4 Physical Medicine and Rehabilitation, University of Michigan, Ann Arbor, MI USA; 5 Department of Biomedical Engineering, University of Michigan, Ann Arbor, MI USA; 6 Robotics Department, University of Michigan, Ann Arbor, MI USA; 7 Michigan Institute for Clinical & Health Research, University of Michigan, Ann Arbor, MI USA; 8 Department of Ambulatory Care Services, Michigan Medicine, University of Michigan, Canton, MI USA; 9 Department of Mechanical Engineering, University of Michigan, Ann Arbor, MI USA; 10 Neuromuscular and Rehabilitation Robotics Laboratory (NeuRRo Lab), University of Michigan, 325 E Eisenhower Parkway, Suite 3013, Ann Arbor, MI 48108 USA

**Keywords:** Hemiparesis, Cerebrovascular accident, Hemiplegia, End-user feedback, Mechanical testing, Walking, Empathy interview, Exoskeleton, Rehab, Mixed methods

## Abstract

**Background:**

Stroke remains a major cause of long-term adult disability in the United States, necessitating the need for effective rehabilitation strategies for post-stroke gait impairments. Despite advancements in post-stroke care, existing rehabilitation often falls short, prompting the development of devices like robots and exoskeletons. However, these technologies often lack crucial input from end-users, such as clinicians, patients, and caregivers, hindering their clinical utility. Employing a human-centered design approach can enhance the design process and address user-specific needs.

**Objective:**

To establish a proof-of-concept of the human-centered design approach by refining the NewGait® exosuit device for post-stroke gait rehabilitation.

**Methods:**

Using iterative design sprints, the research focused on understanding the perspectives of clinicians, stroke survivors, and caregivers. Two design sprints were conducted, including empathy interviews at the beginning of the design sprint to integrate end-users’ insights. After each design sprint, the NewGait device underwent refinements based on emerging issues and recommendations. The final prototype underwent mechanical testing for durability, biomechanical simulation testing for clinical feasibility, and a system usability evaluation, where the new stroke-specific NewGait device was compared with the original NewGait device and a commercial product, Theratogs®.

**Results:**

Affinity mapping from the design sprints identified crucial categories for stakeholder adoption, including fit for females, ease of donning and doffing, and usability during barefoot walking. To address these issues, a system redesign was implemented within weeks, incorporating features like a loop-backed neoprene, a novel closure mechanism for the shoulder harness, and a hook-and-loop design for the waist belt. Additional improvements included reconstructing anchors with rigid hook materials and replacing latex elastic bands with non-latex silicone-based bands for enhanced durability. Further, changes to the dorsiflexion anchor were made to allow for barefoot walking. Mechanical testing revealed a remarkable 10-fold increase in durability, enduring 500,000 cycles without notable degradation. Biomechanical simulation established the modularity of the NewGait device and indicated that it could be configured to assist or resist different muscles during walking. Usability testing indicated superior performance of the stroke-specific NewGait device, scoring 84.3 on the system usability scale compared to 62.7 for the original NewGait device and 46.9 for Theratogs.

**Conclusion:**

This study successfully establishes the proof-of-concept for a human-centered design approach using design sprints to rapidly develop a stroke-specific gait rehabilitation system. Future research should focus on evaluating the clinical efficacy and effectiveness of the NewGait device for post-stroke rehabilitation.

**Supplementary Information:**

The online version contains supplementary material available at 10.1186/s12984-024-01356-3.

## Background

Stroke is the leading cause of long-term adult disability worldwide [1]. By 2030, nearly 4% of the US population is expected to have had a stroke, leading to an estimated cost burden of ∼$184 billion [2]. While some level of spontaneous biological recovery can occur after a stroke, this process is often incomplete, leaving most stroke survivors with persistent gait impairments, which can lead to walking disabilities, falls, and reduced health-related quality of life [3, 4]. As a result, clinicians emphasize the restoration of gait and balance as a central goal of stroke rehabilitation.

**Fig. 1:**
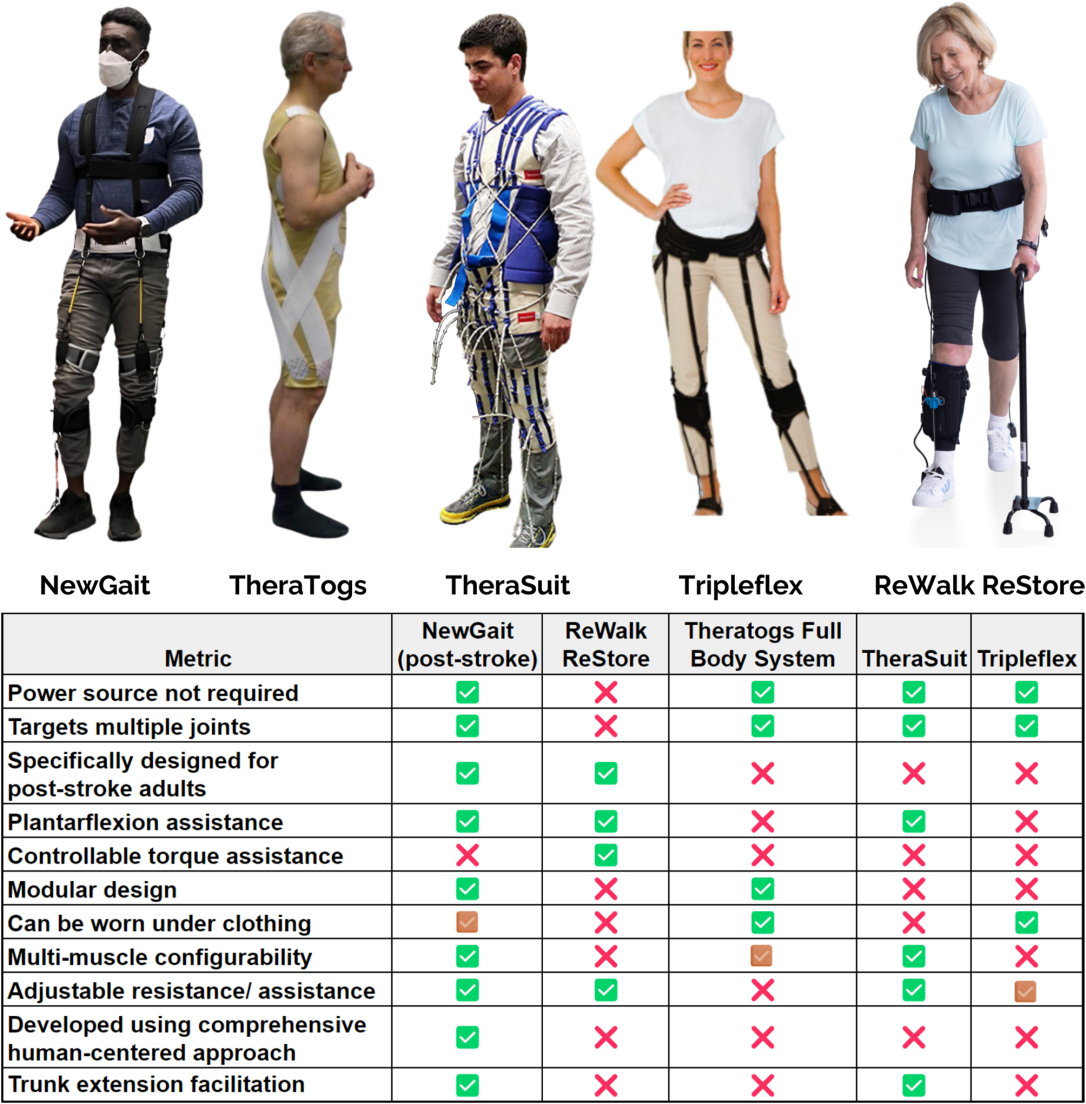
Pictures showing the NewGait device along with other comparable devices such as TheraTogs, Therasuit, ReWalk ReStore, and Tripleflex (Top panel). Table showing a comparative chart of all of the above devices (Bottom panel)

Numerous innovative therapeutic approaches, such as body weight supported treadmill training and robotic therapy, have emerged to address this challenge [5, 6]. However, the outcomes of these interventions have frequently fallen short of expectations, in part due to their high costs and modest benefits, limiting their clinical translation [7, 8]. Recognizing the pressing need for effective, efficient, and low-cost technologies for gait rehabilitation after stroke, researchers and engineers have explored various wearable solutions. Conventional ankle-foot orthoses (AFOs) offer simplicity and affordability, but they may inadvertently result in disuse atrophy and reduced gait efficiency by limiting Achilles tendon excursion and propulsive forces during walking [9, 10]. Moreover, they primarily target the ankle joint, overlooking the essential roles of the hip, knee, and trunk in gait and balance [11]. While devices like TheraTogs [12] and TheraSuit [13] aim to address multiple joint areas, their primary focus on the pediatric market [14] raises challenges when adapting to the adult stroke population (see Fig. 1). TripleFlex [15], a more recent development, targets foot drop and leg-lift deficiencies by putting energy into flexing each joint during the swing phase of gait, thereby offering potential improvements, particularly for adults with neurological conditions like stroke. Additionally, the ReWalk ReStore, while developed specifically for post-stroke gait rehab, only targets the muscles in the shank and requires the user to carry a battery pack on their back [16, 17]. More importantly, most of the existing devices do not offer modularity (i.e., the ability to select and choose different joints) and configurability (i.e., the ability to target different muscle groups) based on patient-specific deficits. Nonetheless, the scarcity of comprehensive studies assessing these devices’ efficacy and effectiveness leaves substantial uncertainties regarding the clinical utility and usability of these devices for stroke rehabilitation.

A recurring issue in the field is that many of these devices have been conceived without adequate input from the individuals who matter most in the rehabilitation process—stroke survivors, their caregivers, and the clinicians providing care. This omission has led to devices that often do not fully align with the actual needs and preferences of these end-users [18–20]. As a result, the utilization of these devices remains limited, and questions linger about how well they address the needs and desires of clinicians and patients, further hindering their clinical utility and usability.

Addressing these challenges necessitates a shift in the approach to device development strategies. Integrating the principles of human-centered design can significantly enhance the development process and result in more inclusive, tailored, and empowering solutions [21, 22]. Human-centered design emphasizes understanding people’s needs, motivations, and concerns while engaging stakeholders from the outset and adopting a systems approach to generalize individual interests to collective solutions [23]. It allows for more effective and efficient design by engaging with users early in the development process, yielding valuable insights while working with prototypes and sketches, rather than fully built products, which can prevent the misallocation of resources [24].

In this study, we employ a novel device design framework based on human-centered design strategies, known as “design sprints” [25], to comprehensively grasp user needs and expectations in order to refine a low-cost, passive exosuit device called NewGait device for stroke rehabilitation [26–28]. The NewGait device (see Fig. 1), originally developed as a sports and performance enhancement device called SpeedMaker®, features lightweight elastic bands, leg straps, a shoulder harness, a waist belt, and movable anchor points for connecting the bands, rendering it highly modular. These elastic bands not only work in concert with muscle and tendon groups to assist or resist motion, but also aid in providing neuromuscular cues essential to neuroplasticity. Additionally, the elastic nature of the bands helps to facilitate proprioceptive feedback for users [29].

While the clinical efficacy of the NewGait device has not been extensively documented, many clinicians have used it to treat gait and balance issues in individuals with stroke and have anecdotally reported noteworthy clinical improvements. However, feedback from clinicians and patients revealed the need for refinement, particularly for stroke-specific populations, as the original device was not intended for this population. Therefore, the purpose of this study was to use human-centered design approaches to tailor the NewGait device to the unique requirements of stroke rehabilitation. We hope that by prioritizing the input and feedback of stroke survivors, caregivers, and clinicians, we will be able to ultimately enhance its clinical usability and effectiveness in improving the lives of those affected by this debilitating condition.

## Methods

### Study overview

**Fig. 2:**
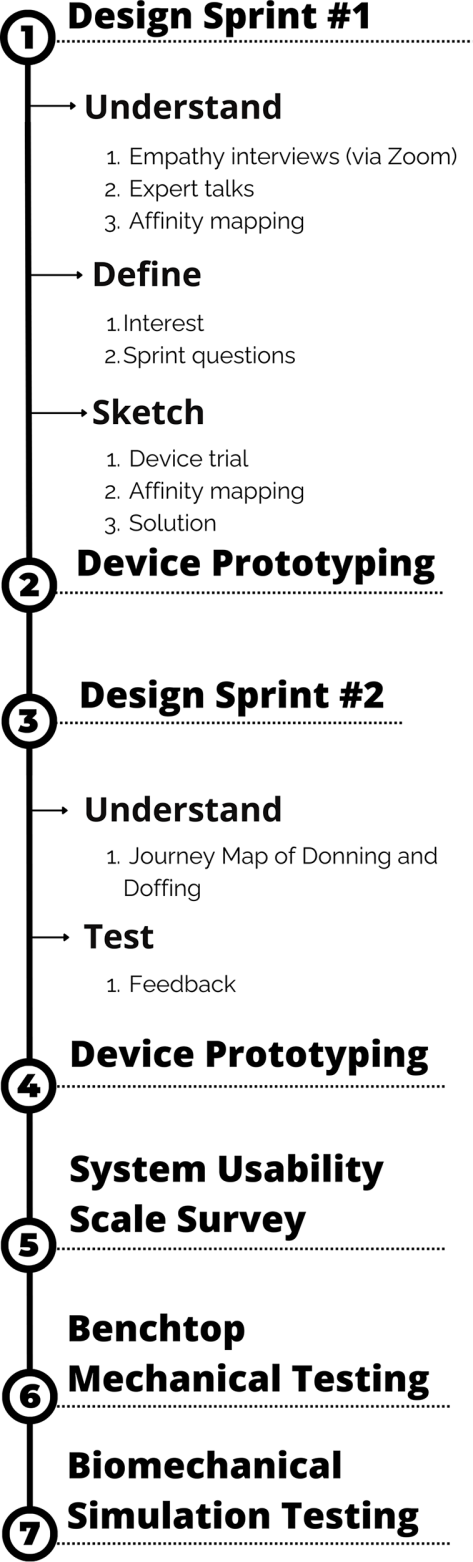
A schematic of the overall study overview

A schematic of the study overview is provided in Fig. 2. Briefly, the study involved (i) two iterations of design sprints (each followed by device prototyping) to develop a stroke-specific NewGait device, (ii) system usability testing in comparison with the older NewGait device and a commercially available competitive device, and (iii) benchtop mechanical testing and biomechanical simulation to evaluate the durability and function of the final prototype.

### Description of the NewGait device

The NewGait is an exosuit designed to enhance functional mobility and correct abnormal gait patterns in individuals experiencing gait and mobility challenges. The NewGait is comprised of a waist belt, shoulder harness, thigh and shank straps, and elastic bands of varying stiffnesses. D-rings are attached to the shoulder harness, waist belt, thigh, and shank straps, and the elastic bands are fitted with safety hooks at each end. The safety hooks on the elastic bands are attached to the D-rings so that the bands span different joints of the user. The bands provide passive elastic resistance or assistance to the joint’s motion, depending on if the motion stretches or relaxes the band, respectively.

The NewGait is unique in comparison to other devices in the market, as it offers several joint articulations, which can each be controlled in a modular and independent manner. For example, bands connecting the harness and waistbelt can maintain upright posture by assisting trunk extension. Similarly, bands connecting the anterior, posterior, and lateral portions of the waist belt and thigh straps can assist hip flexion, extension, and abduction, respectively, and bands connecting the anterior and posterior portions of the shank strap and shoe can assist ankle dorsiflexion and plantarflexion, respectively. The configuration and stiffness selection of bands can be varied to support different joints/phases of the gait based on the user’s needs. More importantly, the NewGait is a fully passive system (i.e., no electrical components) and is manufactured from neoprene, rubber, and nylon, making it lightweight and portable.

### Design sprints

*Design sprints* attempt to compress the human-centered design process into a compact schedule of several hours [30]. A traditional design sprint, as initially formulated by Google Ventures, lasts five days, with each day dedicated to one of the five stages [25]:

1. *Understand (and Define)*: Identify and gain insight into the problem.
2. *Sketch*: Explore solutions to the problem.
3. *Decide*: Survey proposed solutions and select which will be incorporated into prototype.
4. *Prototype*: Incorporate selected solutions into device to create new prototype.
5. *Test*: Stakeholders use new prototype and provide feedback.

**Fig. 3:**
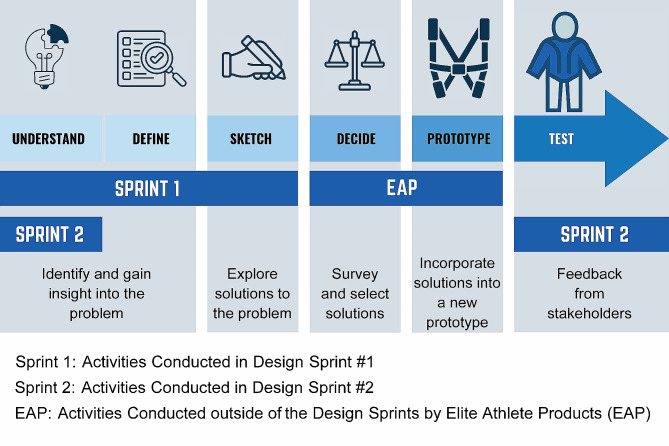
A schematic of the design sprint overview

The design sprint process encourages teams to fail faster, meaning that shortcomings in a product or process are brought to light early in the design process before significant time and money have been invested. Ideally, the participants in a sprint bring varied perspectives on the problem, for example, clinicians, patients, caregivers, designers and/or programmers. Because the NewGait device is a pre-existing product and not being created from scratch [26], the design sprint process was slightly modified and consisted of two design sprints, each conducted in a single day for a duration of seven hours (Fig. 3). The first design sprint (Design sprint #1) focused on the *Understand, Define*, and *Sketch* stages. The *Decide* and *Prototype* steps were executed externally by the NewGait team following each design sprint. Specifically, the NewGait device technical team made necessary adjustments to improve the prototype to address key issues and recommendations that emerged from each design sprint. The second design sprint (design sprint #2) focused on the *Understand* and *Test* stages. Once the product development was complete, benchtop mechanical testing and biomechanical simulations were conducted to evaluate the durability and function of the new NewGait device. Additionally, a system usability evaluation was performed to compare the usability of the newer prototype with both the older version of the NewGait device and a competitor’s device (TheraTogs). These evaluations provided valuable data on product durability, as well as usability in comparison with existing solutions.

All study activities were approved by the University of Michigan IRBMED as exempt; participants provided verbal agreement and were offered $50 for their participation.

### Design sprint #1

Design sprint #1 stared with user interviews (i.e., empathy interviews) during the *Understand* phase to gain insights from stroke patients and incorporate their perspectives into the development process [31]. Unlike traditional design sprints, these empathy interviews happened on separate days via Zoom to ensure that the onsite design sprints could be concluded in a timeframe that is more tolerable to stroke survivors. The primary goal for empathy interviews was to engage clinicians with expertise in providing physical or occupational therapy and stroke survivors to learn more about the selection, adoption, and utilization of mobility aids. Specifically, interviews focused on the benefits of mobility aids, preferences around single use devices or a multi-functional device, features that make a mobility device acceptable, and strategies to facilitate the selection, adoption, and utilization of a mobility aid. Results of the empathy interviews helped to determine the constraints of the design sprints and where input would be most useful, which was particularly important given the NewGait device is an existing product. All interviews were moderated by an expert in design sprints (MB) and an expert in rehabilitation (CK); all interviews were audio-recorded and professionally transcribed.

During the onsite design sprint #1, we followed the traditional *Understand* process, using notes taken by participants during *Expert Talks* to identify important themes. *Expert Talks* allow each of the participants to explain their unique perspective on the design problem [30]. Although each conversation takes only 5–10 min, the questions the facilitators ask (MB and CZK) are designed to give the participants an opportunity to discuss their knowledge, feelings, and experiences. During these conversations, the other participants take note of ideas that surprise, intrigue, or resonate with them. Participants share the notes that they took from those conversations with the entire group. These notes form the raw material for the subsequent *Affinity Mapping* step. Affinity mapping in design sprints is a tool for organizing complex sets of data into meaningful categories, enabling a user-centered approach to design by highlighting user needs and preferences. To form affinity maps, participants use the notes taken during the expert talks and arrange them into common themes through discussion. This method helps teams move from a broad range of ideas to focused, actionable insights.

After discussing these themes in the group, a single theme is given priority (*Define a Sprint Question*) to focus the work of the rest of the session. With this theme in mind, participants then used the NewGait device (as a modified *Sketch*). Specifically, the stroke survivors were fitted with the device and then moved naturally around the space. The participants ambulated on level ground, completed transitional movements such as sit to stand transfers, and ascended and descended a staircase, while other sprint participants took note of their feedback. The participants who were fitted with the device then gave feedback on their experience. We then identified the themes present in this user feedback, deciding on what the participants felt were the most pressing issues. Six participants were involved in design sprint #1: NewGait developer (OPA), three rehabilitation researchers who are rehabilitation engineers, and two stroke survivors. The sprint was led by an expert in user-centered design (MB) and human-centered research (CZK). A postdoctoral fellow assisted stroke survivor participants with notetaking.

### Between design sprints

After design sprint #1, the NewGait team reviewed the Affinity Maps of feedback based on end-users trying the NewGait device and decided (*Decide* phase) what to incorporate into a new prototype (*Prototype* phase) device, which was used in Sprint #2. Whereas these steps often co-occur during a single design sprint, due to the technical knowledge needed to make appropriate adjustments to the device, the NewGait team evaluated and adopted what they felt were appropriate modifications to the device based on the user input from design sprint #1.

### Design sprint #2

**Fig. 4:**
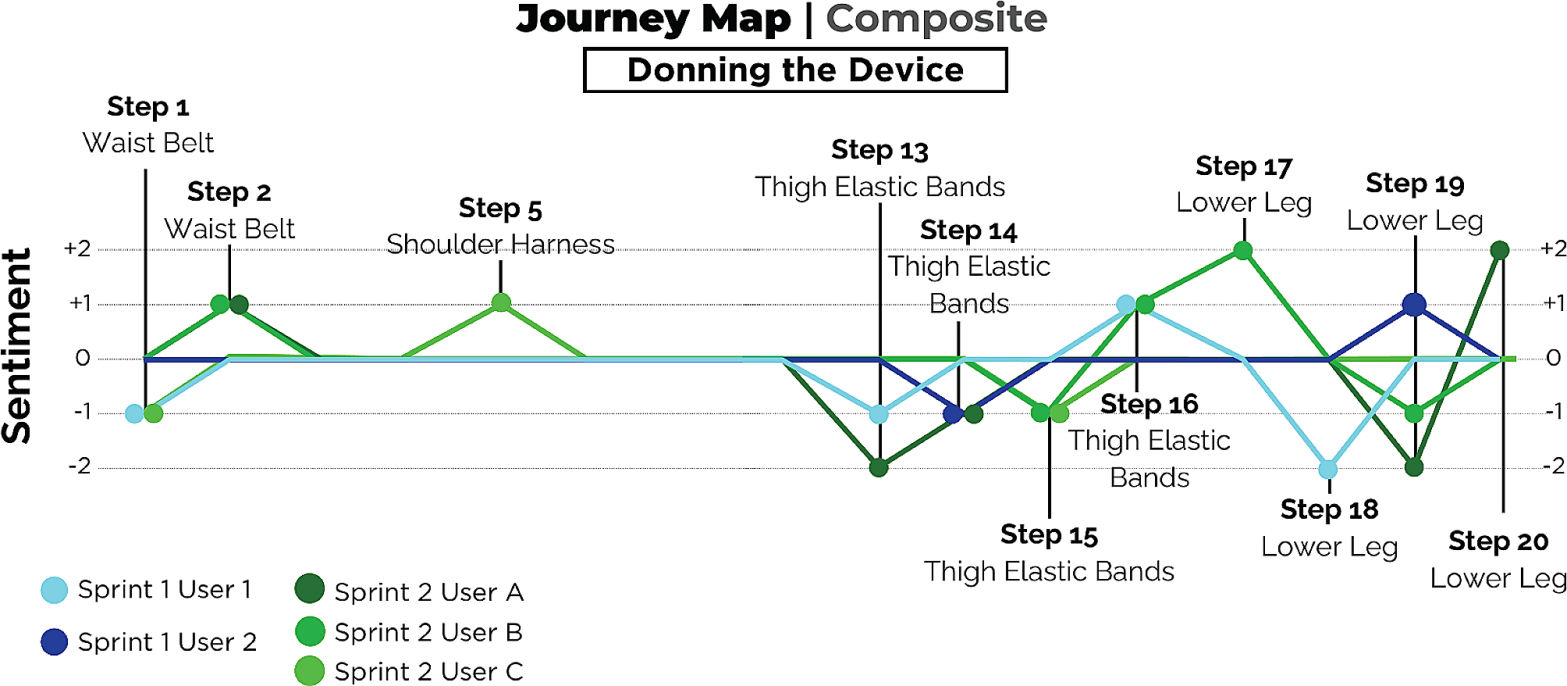
A schematic of the device donning journey map from design sprint #1 and #2

**Fig. 5:**
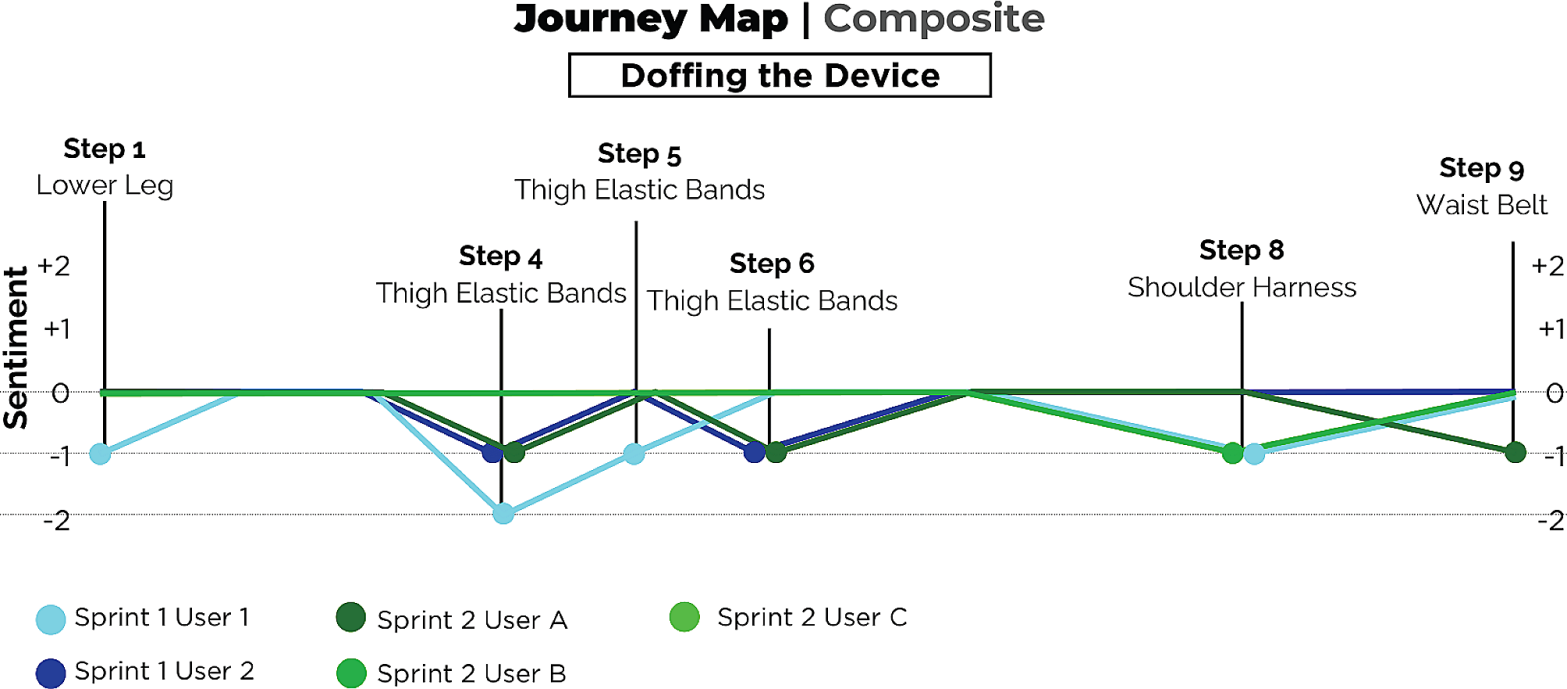
A schematic of the device doffing journey map from design sprint #1 and #2

In design sprint #2, we used the *Understand* phase to construct a Journey Map of the donning and doffing process for the device, which was one of the major themes that emerged from design sprint #1 (see results section for details). A Journey Map is a visualization tool that depicts a user’s experience as they attempt to accomplish a goal [32]. A Journey Map typically includes two parts: a step-by-step breakdown of the process being evaluated, and the sentiment (or pain points rated between − 2 and + 2) associated with each step. As a group, the participants helped break down the donning and doffing processes into the component steps (see Supplementary Material). Each of the three stroke survivors was matched with a clinician with experience using the NewGait device who helped fit the refined prototype based on their specific needs. Other design sprint participants took note of their feedback as they moved around with the device, organizing the feedback into positives (+), negatives (−), questions (?), and new ideas (!) (see Supplementary Material). As the users then tested the new prototype (*Test* phase), this feedback was then matched with the Donning and Doffing Journey Maps (see Figs. 4 and 5; see Supplemental Materials for individual journey maps) to match each user’s emotional response to the different steps (i.e., visualize pain points in the process that were worth addressing). Results of empathy interviews from Design sprint #1 also informed the Journey Maps. Eleven participants were involved in design sprint #2: NewGait developer (OPA), two rehabilitation researchers who are rehabilitation engineers, two physical therapists and one prosthetist who were experienced users of the NewGait device, and three stroke survivors and two caregivers. The sprint was led by the same expert in user-centered design (MB) as for design sprint #1. A postdoctoral fellow assisted stroke survivor participants with notetaking.

### Post-sprint feedback

Upon the culmination of the two design sprints, we implemented design modifications to the NewGait device. We then administered System Usability Scale surveys (SUS) to patients and physical therapists to assess the usability of the original device, the new prototype, and a competitive device (Theratogs®, Telluride, Colorado). The System Usability Scale (SUS) [33] is a reliable and popular measure of a user’s perception of the usability of a device, software, or system. It is a 10-item questionnaire with five response options from Strongly agree to Strongly disagree. The final score is converted to a scale, ranging between 0 and 100, where a higher score indicates greater usability. The final scores can also be converted into a percentile score to better interpret the usability of the system. The interpretation of the scores of the SUS surveys are as follows: 84.1–100 indicates the “Best Imaginable” score, putting the device in the 96-100th percentile, 80.8–84.0 indicates an “Excellent” score, putting the device in the 90-95th percentile, 62.7–80.7 indicates a “Good” score, putting the device in the 35-89th percentile, and a score below 51.7 indicates an “Okay” score, putting the device below the 35th percentile (see, Supplementary Material).

### Durability testing

**Fig. 6:**
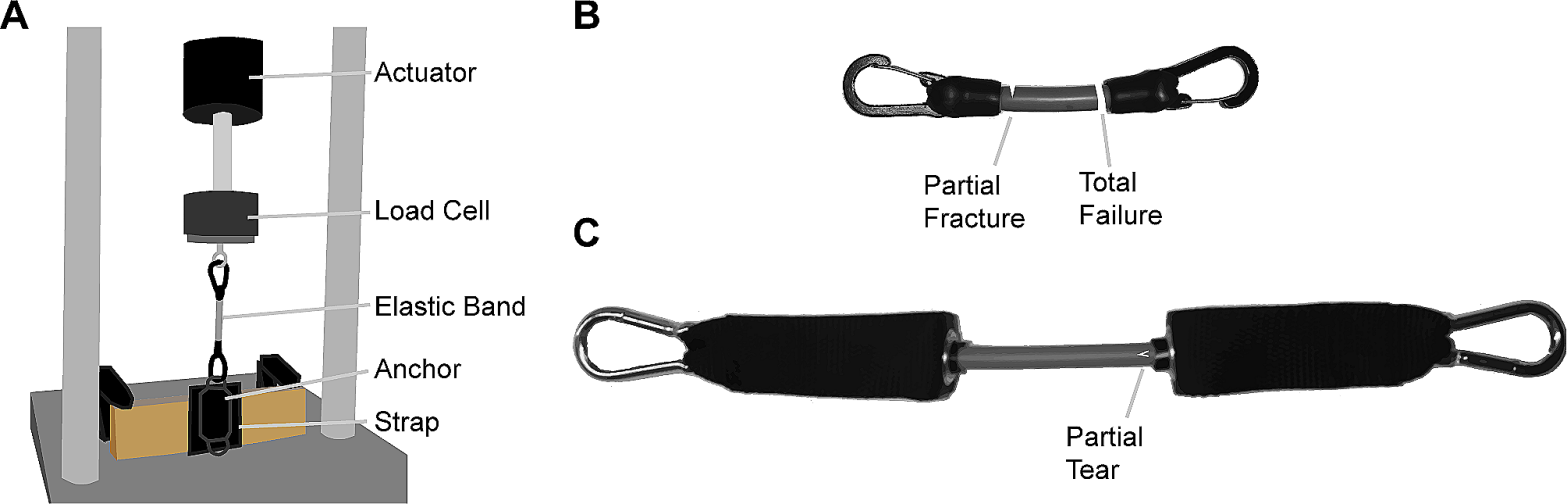
Durability testing. **(A)** Schematic of the system used for durability testing of the NewGait prototype design. **(B)** Representative schematic indicating the failure zones on the latex elastic band after total failure (26,727 cycles). **(C)** Representative schematic indicating the condition of the silicone elastic band after completing 500,000 cycles of loading

Durability (i.e., fatigue) testing was performed to ensure that the new prototype could withstand repeated loading that would be expected while using the device (Fig. 6A). Fatigue testing was performed on the interfaces between the straps, anchor points, and elastic bands, which we identified as the likely failure points. We tested elastic bands made of two materials of similar stiffness: latex-based, as has been used with previous iterations of the NewGait device, and silicone-based, which are known to be more durable. These device components were loaded into a hydraulic tensile testing machine (Instron 8521, Canton, MA, USA). Briefly, a hook-and-loop neoprene strap was fastened to a wooden block that was clamped down to the base of the tensile testing machine. An anchor point was attached to the strap and the elastic band was connected between the anchor point and the actuator of the tensile testing machine. The cross-head of the machine was adjusted so that the band was not in slack when the actuator was fully extended. Care was taken to ensure proper alignment of the sample with the actuator. The machine was configured so that the components would undergo 10 cm of deformation in accordance with a sine wave (frequency = 1.75 Hz). The 10 cm of deformation was based on band excursions obtained from biomechanical data of stroke survivors. Note that although the silicone tubing had a longer interface with the clips, the active elastic portion of each band was the same length. The characteristics of the loading (i.e., force, deformation, and rate) were recorded using on-board instrumentation. The sample was repeatedly loaded until either the sample failed (e.g., breaking of the elastic band or degradation of the hook and loop fastening), or the machine reached 500,000 loading cycles (equivalent of 3000 steps/day for 6 months, as anticipated for clinical or in-home use).

### Biomechanical simulation

To indicate how various NewGait configurations can be used to alter muscle activation, we ran a simulation in OpenSim (version 4.4) using data from a stroke survivor (sex: male, age: 73 years, height: 168 cm, walking speed: 0.85 m/s, Lower-Extremity Fugl-Meyer: 26). Prior to participation in this study, the participant signed an informed consent document approved by the Wayne State University Institutional Review Board. To obtain data for the simulation, the participant walked overground while wearing the straps and D-rings of the NewGait device (no bands were placed on the device). The walkway was 6 m long and embedded with a force plate (5060-06, Bertec, Columbus, OH, USA). Lower-extremity kinematics were recorded using a 10-camera motion capture system (Vicon Vero v2.2). Reflective markers were placed over the anatomical landmarks of the subject’s lower extremity, with clusters placed on the thigh and shank. Additionally, markers were placed over the D-rings of the NewGait device to indicate the location where bands could be anchored. We also collected a static trial with the subject standing upright in a neutral posture.

To run the simulation, the motion capture data were exported into OpenSim. Using the Scale Tool and the marker data from the static trial, we scaled a generic musculoskeletal model (gait2392) to match the anthropometry of the stroke patient. Using the Inverse Kinematics Tool and the marker data collected during walking, we calculated the joint angles of the participant as they walked. Finally, using the Static Optimization Tool with the kinematics and an external force file (i.e., ground reaction forces), we calculated the muscle activation of several key muscles in the model as it walked. Prior to running static optimization, the strength of the muscles in the scaled model was increased by 25% to ensure that the muscle activation did not exceed the maximum level when calculating muscle activation. Reserve actuators were placed on all degrees of freedom for the pelvis.

To simulate the effects of walking with the NewGait, the marker positions corresponding to the device’s anchor points were imported into MATLAB (R2021b, Mathworks, Natick, MA, USA). The distance between these marker positions was calculated during walking, and we calculated the force that would result from a blue resistance band (length: 107 mm, stiffness: 210 N/m) connected at these positions. We calculated the resulting forces from five NewGait configurations with bands overlaying the: (1) hip flexors and ankle dorsiflexors (2), hip extensors and ankle dorsiflexors (3), hip extensors and ankle plantarflexors (4), hip flexors, hip abductors, ankle dorsiflexors, and (5) hip extensors, hip abductors, and ankle dorsiflexors. For each condition, the resulting band forces were added to the external force file, with counteracting forces fixed to the proximal and distal segments of the model. We then ran static optimization for each configuration using this updated external force file.

For each NewGait configuration and the normal walking trial, the muscle activation of the rectus femoris (RF), vastus medialis (VM), sartorius (St), biceps femoris long head (BFL), gluteus maximus (GMax), tibialis anterior (TA), medial gastrocnemius (MG), soleus (Sol), and gluteus medius (GMed) muscles were measured over the gait cycle. The gait cycle data were averaged into bins corresponding to distinct phases of gait, including the loading response (LR), mid stance (MSt), terminal stance (TSt), pre-swing (PSw), initial swing (ISw), mid-swing (MSw), and terminal swing (TSw). The normal walking muscle activation data were subtracted from the NewGait data and depicted in heatmaps to indicate phases of the gait cycle where the muscles either increased or decreased in activation.

## Results

### Participants

**Fig. 7:**
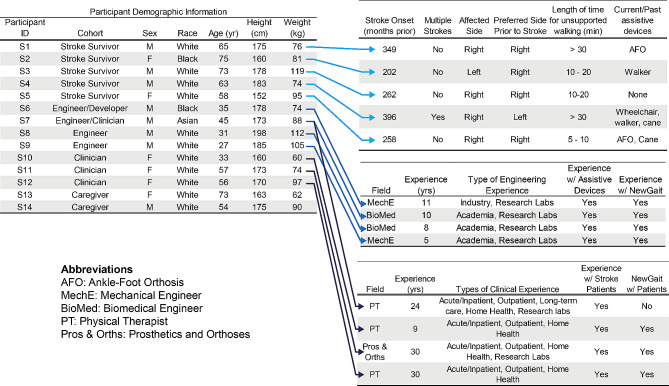
Demographic information of design sprint study participants. (Left) Basic demographic information for all participants. (Right) Cohort-specific demographic information

Participants in empathy interviews included four stroke survivors, two occupational therapists, and two physical therapists. One interview included both stroke survivors and their physical therapist. Fourteen unique adults participated in the design sprints: Six adults participated in design sprint #1, and eleven adults participated in design sprint #2 (Fig. 7). Participants were eligible to participate if they belonged to one of the following cohorts: stroke survivor, caregiver of a stroke survivor (e.g., spouse), clinician, or engineer.

### Empathy interviews

Interviews highlighted the common patient frustrations using aids that are heavy or uncomfortable, leading to their eventual abandonment. For both clinicians and stroke survivors, usability factors such as donning or doffing as independently as possible, were critical in adoption and utilization. Features like being lightweight and easy to put on one-handed, offering support and stability were noted as particularly important. The visual appearance of an aid while wearing could also be relevant, particularly for initial adoption. High costs were generally prohibitive for most users in purchasing aids out of pocket.

### Design sprint #1 – affinity mapping

**Fig. 8:**
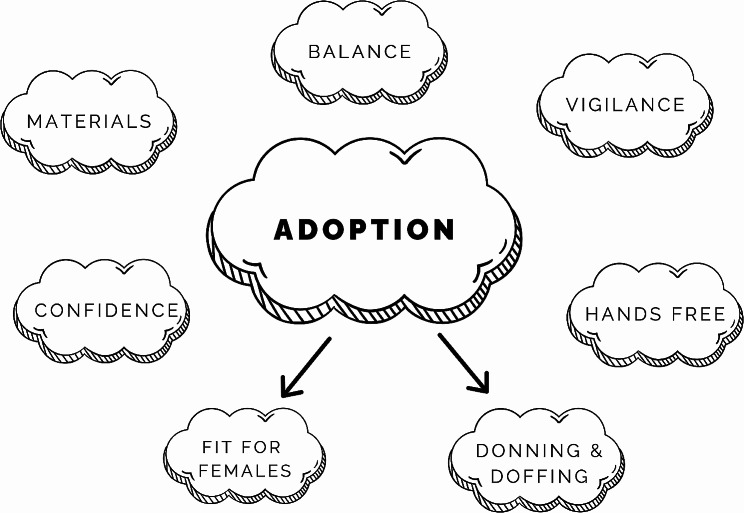
A schematic of affinity mapping themes from design sprint #1. Note that upon reviewing the themes from the affinity mapping process, participants felt that “Adoption” was the most important to investigate further for the stroke-specific NewGait device. Once the users had the opportunity to use the device in a variety of positions and have their feedback recorded, the group took that feedback and grouped the notes by themes. After discussing the relative importance of the themes that emerged under “Adoption”, the group agreed that “Donning & Doffing” and “Fit for Females” were the two most important issues to be addressed further

The themes that resulted from the Experts Talks and Affinity Mapping Process are shown in Fig. 8 (also see Supplementary Material). In reviewing these themes, the participants felt that “Adoption” was the most important theme that needed to be investigated further. Once the stroke survivors had the opportunity to use the NewGait device in a variety of configurations and have their feedback recorded, the group revisited their previous discussions and agreed that “Donning & Doffing” and “Fit for Females” were the two most important themes that needed to be addressed in the new prototype (Fig. 8).

### Between design sprints

Substantial modifications were identified and implemented between the first and second design sprints to address the themes identified in Design Sprint #1. In the context of the donning and doffing area, the NewGait team transitioned the device to loop-backed neoprene for the primary components of the system, facilitating enhanced adhesion of any hook component to the device. The shoulder harness was reengineered, featuring a novel closure and attachment mechanism to the waist belt. This revision entailed replacing the traditional buckle system with a hook and loop design, thereby simplifying adjustment and donning processes. Additionally, the material of the shoulder harness keeper was upgraded from flexible ethylene vinyl acetate material to a more robust low density polyethylene (LDPE), capable of resisting deformation under the stress exerted by elastic bands.

**Fig. 9:**
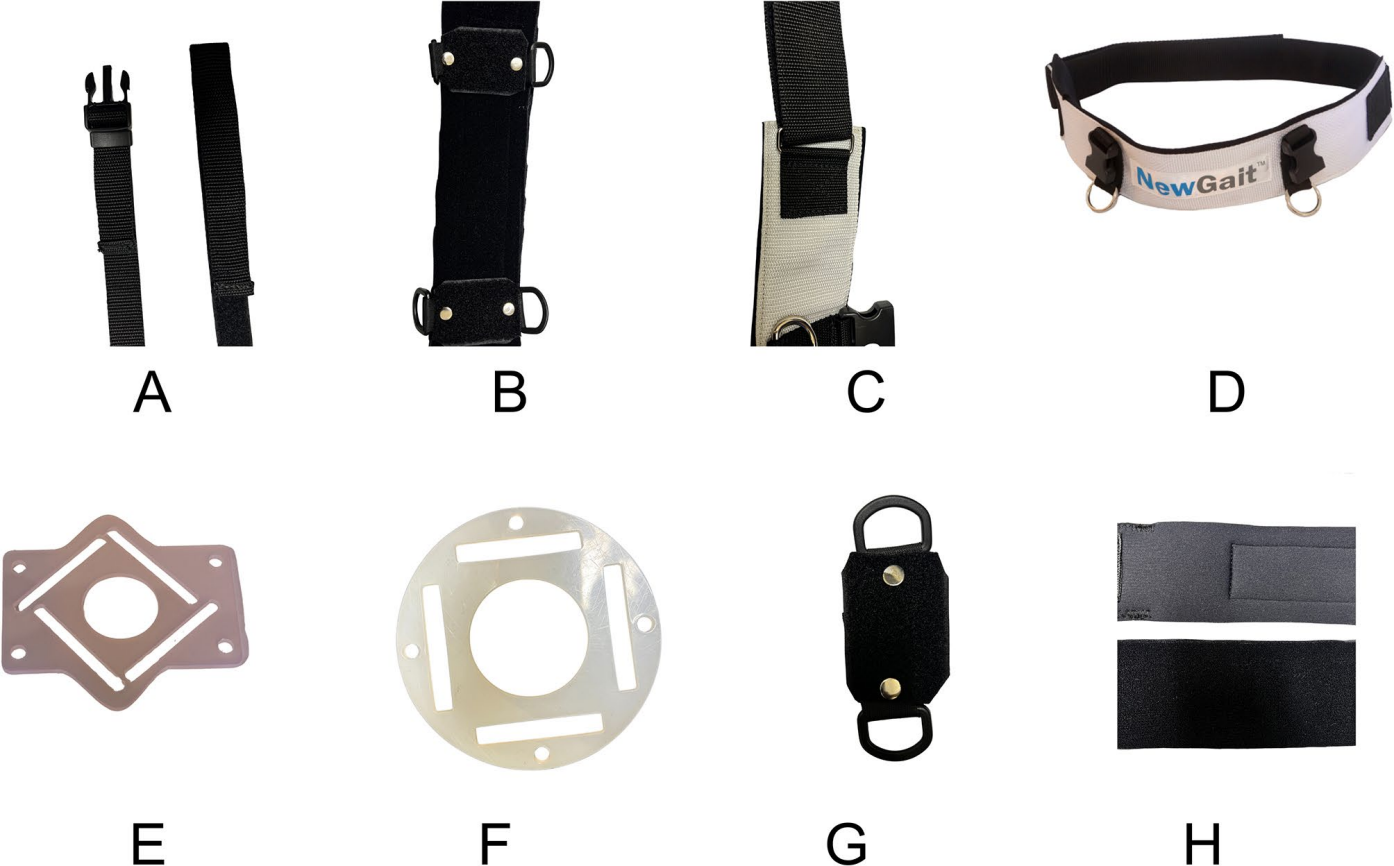
The specific design changes made after design sprint #1. **(A)** the traditional buckle style clip (left) and the updated hook and loop connection (right) that connects the shoulder harness and waist belt. **(B)** The updated wait belt with two adjustable anchor points. These anchors can be adjusted laterally to accommodate different sized users. **(C)** The original waist belt pullback strap closure mechanism of the older version of the device. **(D)** The original style waist belt showing the two fixed anchor points on the frontside. **(E)** The original shoulder harness keeper made from Flexible EVA (ethylene vinyl acetate). **(F)** The newly designed shoulder harness keeper made from a more robust low density polyethylene (LDPE). **(G)** The newly design anchor, made using opposing hook and loop material on either side. **(H)** The original neoprene used throughout the device has a nylon layer. (top) The newly designed prototype utilizes a neoprene with loop material (bottom)

The waist belt also underwent extensive redesign. The closure mechanism was transformed from a pullback strap to a straightforward hook and loop system. The anchors connected to the waist belt were reconstructed using rigid hook materials, enhancing their mobility around the waist belt and leg straps. This modification also facilitates lateral adjustments of the connection point between the shoulder harness and waist belt, accommodating users with varying breast sizes and shapes (Fig. 9).

Collectively, these design alterations resulted in a product with reduced stitching, potentially increasing durability. The new design was less cumbersome and offered improved discretion when worn under clothing. These advancements collectively herald a design that not only meets functional requirements but also accommodated a wider range of body types, all while maintaining discretion and comfort when worn beneath clothing.

### Design sprint #2 – journey mapping

**Fig. 10:**
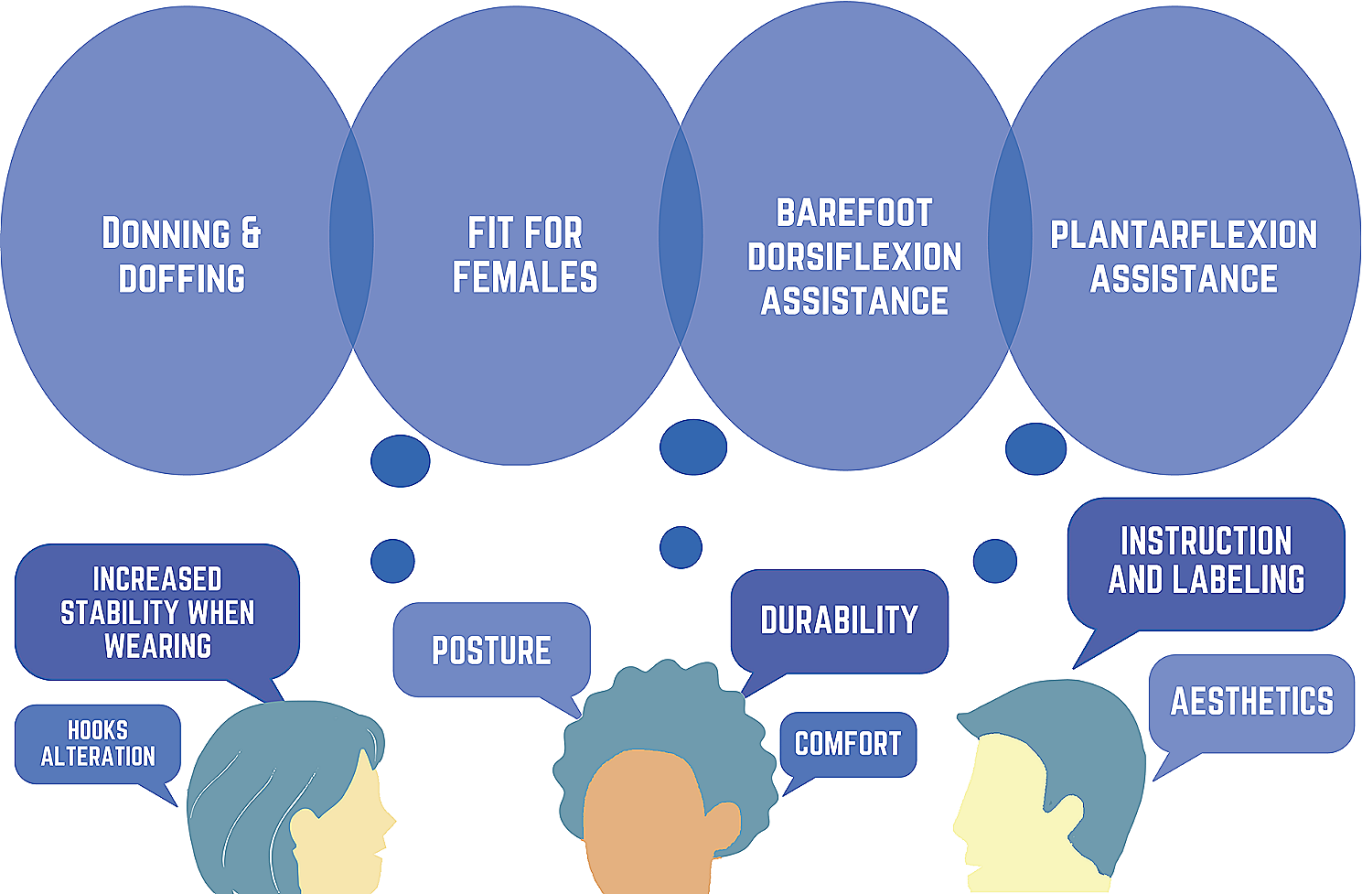
A schematic of the primary themes that emerged from design sprint #1 and #2. Note that “donning and doffing” and “fit for females” emerged from design sprint #1 and “barefoot dorsiflexion assistance” and “plantarflexion assistance” emerged from design sprint #2

At the start of design sprint #2, the NewGait team (OPA and two therapists) modeled the new prototype device. The journey mapping process of donning revealed that stroke survivors appreciated the positive impact it had on their posture and foot clearance. However, they also mentioned the need for multiple adjustments to achieve an ideal fit. On the other hand, the journey mapping process of doffing highlighted that, while stroke survivors noticed an appreciable improvement in stability and posture upon removal, they encountered difficulties in managing the hook and loop components, often struggling to keep them from sticking together inadvertently. The journey map and feedback from the device test phase indicated the need for adding plantarflexion assistance and barefoot dorsiflexion assistance capability to the NewGait stroke-specific device (Fig. 10).

### Post-sprint design changes, feedback, and durability testing

Following the second design sprint, the team implemented modifications concerning the elastic band material driven from design sprint # 1, and attachment points of the elastic bands to the shoe/foot area, driven by feedback from design sprint # 2 to allow for barefoot walking and enhanced plantarflexion assistance. We replaced the latex-based elastic bands with silicone-based (latex-free) elastic bands, as the silicone-based elastic bands may be a more durable option for the NewGait device training. While silicone is a more expensive material, changing the bands to silicone could permit cost savings for patients and clinicians, as they would have to purchase fewer bands for routine treatment. Latex is also known to degrade more than silicone due to environmental factors [34]. Silicone also has the added benefit of being hypoallergenic, as latex allergies are common [35].

**Fig. 11:**
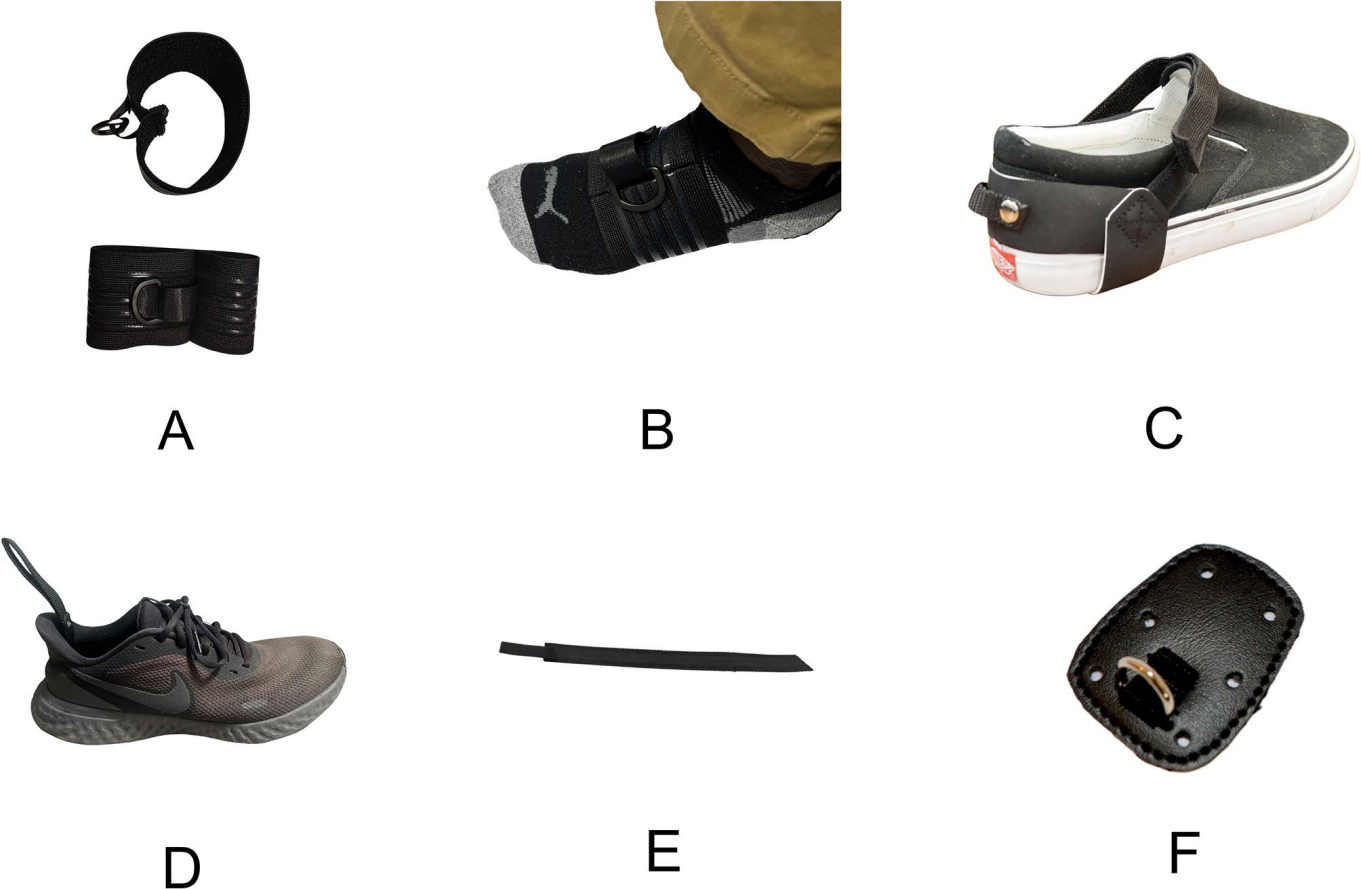
The specific design changes made after design sprint #2. **(A)** The barefoot dorsiflexion anchor. **(B)** The barefoot dorsiflexion anchor on a foot. **(C)** The over-the-shoe plantarflexion anchor. **(D)** The in-the-shoe plantarflexion anchor shown in a shoe. **(E)** The in-the-shoe plantarflexion anchor shown laid out. **(F)** The original lace-in dorsiflexion anchor of the older NewGait version

For barefoot walking, the modification entailed the development of a dorsal attachment piece to aid in dorsiflexion. This was achieved using an elastic material featuring a d-ring, designed to comfortably fit over the user’s foot without the need for shoes. For enhanced plantarflexion assistance, we developed two new straps to be used inside and outside a user’s shoe. The first strap was engineered to be secured inside the shoe, beneath the sole. This strap included a small loop protruding 1–2 inches past the heel, aligning with the Achilles tendon. The second strap introduced a strap assembly that wraps around the shoe, equipped with a loop near the heel for carabiner attachment, thus aiding in plantarflexion (Fig. 11).

To address donning and doffing issues (e.g., inability to correctly doff such that the hook and loop components do not inadvertently stick to each other) that were pointed out during the Design Sprint #2 Journey Mapping, we developed user manuals and tutorials that would improve the usability of the device. Results of the SUS revealed that Theratogs® scored an average of 46.9, the original NewGait device achieved 62.7, and the new stroke-specific NewGait device obtained a score of 84.3 in the usability assessments. A SUS score above 84.1 would be considered the best imaginable and equivalent to a 96 percentile (see Supplementary Material).

Durability testing of the updated NewGait device was performed over weeks. The updated hook and loop neoprene straps and accompanying anchor points withstood testing and did not show any signs of deterioration with repeated loading. The elastic bands proved to be the weak point of the system. Latex-based bands were unable to withstand the number of cycles that would be required for routine use of the device and failed after 26,727 cycles. Inspection of the sample indicated that failure occurred at the interface between the elastic tubing and the clip, and partial fractures could be seen along the length of the band (Fig. 6B). Silicone-based elastic bands proved to be much more durable and remained intact after 500,000 cycles with just a small tear near the interface of the elastic tubing and the clip (Fig. 6C).

**Fig. 12:**
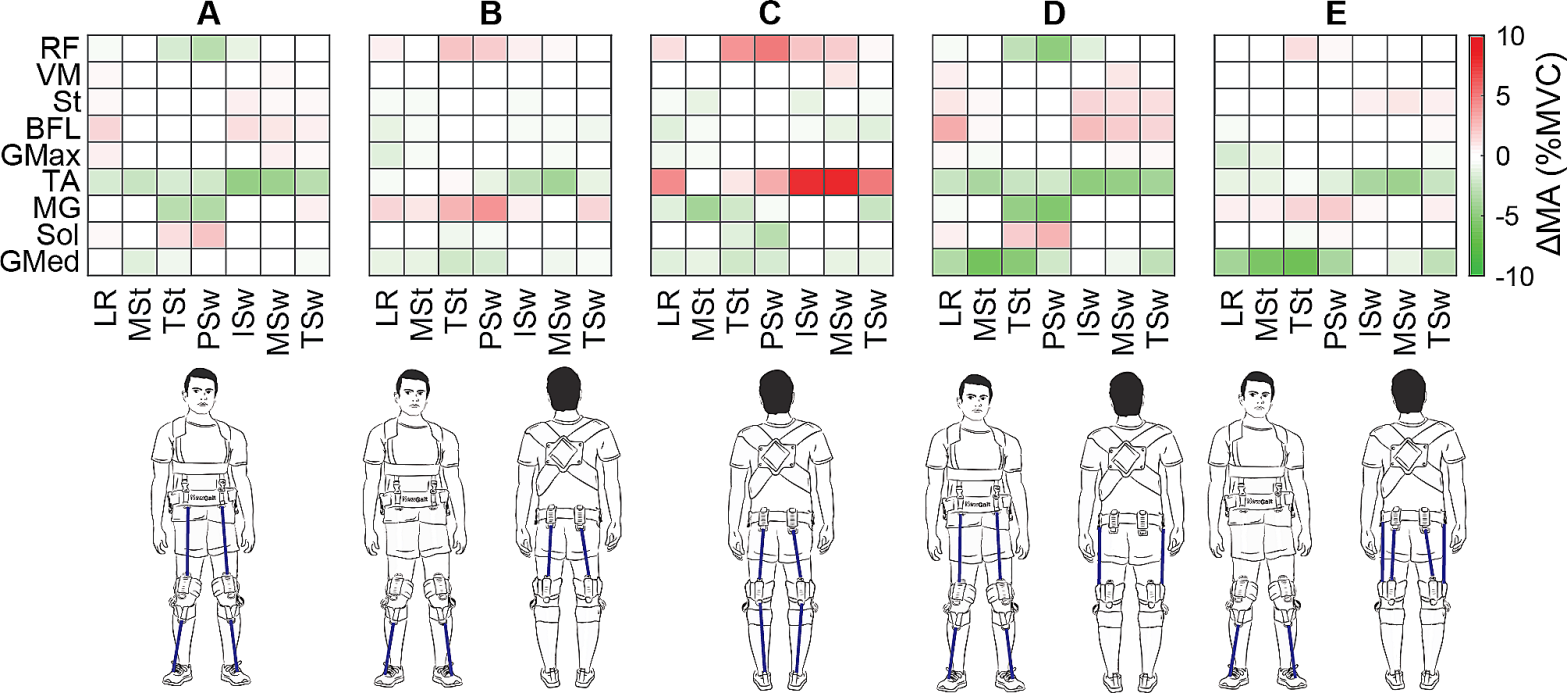
Heatmaps showing changes (Δ) in lower extremity muscle activation data obtained from stroke-specific OpenSim biomechanical simulation using various configurations of the NewGait stroke-specific device. Bands placed overlaying **(A)** hip flexors and ankle dorsiflexors, **(B)** hip extensors and ankle dorsiflexors, **(C)** hip extensors and ankle plantarflexors, **(D)** hip flexors, hip abductors, and ankle dorsiflexors, and **(E)** hip extensors, hip abductors, and ankle dorsiflexors. Changes in muscle activation were computed by subtracting activation data during normal overground walking without the NewGait device from activation data during overground walking with the simulated NewGait device. Green on the heatmaps indicate a reduction in muscle activation (assistance) whereas red on the heatmaps indicate an increase in muscle activation (resistance). ***Abbreviations***: RF: rectus femoris, VM: vastus medialis, St: sartorius, BFL: biceps femoris long head, Gmax: gluteus maximus, TA: tibialis anterior, MG: medial gastrocnemius, Sol: soleus, GMed: gluteus medius, LR: loading response, Mst: mid stance, TSt: terminal stance, PSw: pre-swing, ISw: initial swing, MSw: mid-swing, and TSw: terminal swing

Biomechanical simulation established that different configurations of the NewGait device could be used to selectively assist or resist different muscle groups during walking (Fig. 12). For example, elastic bands placed over the hip flexors and ankle dorsiflexors reduced tibialis anterior activation throughout the gait cycle, while increasing soleus activation and decreasing medial gastrocnemius muscle activation during terminal stance and pre-swing. This configuration also assisted during swing phase by reducing rectus femoris and gluteus medius muscle activation. In contrast, elastic bands placed over the hip extensors and ankle dorsiflexors, increased medial gastrocnemius and rectus femoris muscle activation throughout the gait cycle, while reducing tibialis anterior and gluteus medius muscle activation. The detailed effects of different band configuration on lower extremity muscle activation during different phases of the gait are depicted as heat maps in Fig. 12.

## Discussion

In this study, we developed a refined version of the NewGait device for post-stroke rehabilitation through iterative design sprints, involving essential stakeholders: patients, caregivers, and clinicians. To our knowledge, this is the first exosuit device that was developed using a human-centered design approach that involved iterative design sprints for post-stroke gait rehabilitation. This iterative methodology was pivotal in developing a product that is both user-friendly and clinically adoptable, reflecting the core values of human-centered research. Through this process, we gathered critical feedback on the pros and cons of the existing NewGait device and the refined prototype, which played a significant role in refining the product’s usability and enhancing the overall user experience. The implementation of this structured approach resulted in effective stakeholder participation and the development of a superior product (improved usability, comfort, modularity, and durability).

We believe that the changes made to the design of the NewGait device through design sprints were markedly more efficient compared to a traditional non-structured approach. This is because the speed at which we were able to develop the final stroke-specific prototype (about 3 months) was several folds faster than our previous devices, which took years to develop. A key reason for this efficiency is due to the concentrated collaboration with diverse stakeholders within a structured environment, which significantly accelerated the design process. The result was not only a faster development timeline but also a more refined product achieved through an efficient and systematic methodology. This was supported by the results of the SUS survey scores where clinicians and patients unanimously reported substantial improvements in system usability of the new prototype in comparison with the older version and a competitive product.

The new design boasts improved modularity and flexibility, expanding its application scope. Separately, enhancements in the design have also led to increased durability and cost-effectiveness because the newer prototype requires less stitching and calls for fewer product stock keeping units. The design’s comfort level was also enhanced, which is anticipated to improve user compliance. These features are especially relevant in achieving our overall goal of creating a more accessible, low-cost post-stroke gait rehabilitation device. The overall improvements in the device are expected to promote greater independence and well-being for users, thereby widening its global impact and applicability.

The stroke-specific NewGait device is not the only choice stroke survivors have to rehabilitate from gait and balance impairments. Many devices exist, from textile-based passive devices to electro-mechanical robotic suits, but none have extensive research performed on the post-stroke population. For example, TheraTogs is a device worn directly on the skin, making it difficult to use in a clinical setting and/or with multiple patients, and there is limited research for post-stroke rehabilitation using the device. Other competitive devices, such as Axiobionics Tripleflex, and Therasuit, address the limitation of TheraTogs, but were not developed using a rigorous human-centered approach and have no published research on post-stroke rehabilitation. The ReWalk ReStore exosuit is a robotic device that has been specifically designed and tested for post-stroke rehabilitation and has had extensive research performed. Results have shown improved walking speed (10 MWT) and endurance (6-minute walk test) [36]. However, in a multi-site clinical trial of the safety, reliability, and feasibility of the device, end-user survey responses were mixed. Moreover, the cost of this device is prohibitively expensive to be widely adopted by patients and clinicians for stroke rehabilitation. User adoption is critical for any new product, which is why the stroke-specific NewGait was designed with feedback from multiple stakeholders. Preliminary studies [26–28] and anecdotal experience from clinicians and stroke survivors indicate that the NewGait device has significant clinical potential because of its modularity and configurability for patient-specific deficits, which was also supported by our biomechanical simulation results. While the clinical effectiveness of the NewGait device for post-stroke rehabilitation is currently unclear (this is beyond the scope of this study), future studies will be performed to address this gap.

### Strengths and limitations

A particular strength of our approach to this work was the engagement of all study team members with diverse backgrounds and experience in interpreting feedback gathered in all phases of this study. In doing this work, it is important to avoid two pitfalls. The first is the inevitable desire for overvaluing positive feedback and undervaluing negative feedback. Because we were invested in the success of our efforts to modify the device to meet the needs of stroke survivors, the risk of this bias influencing our interpretation of feedback is higher than it may be with quantitative measures. The second pitfall is the tendency of groups to fall into “group think” and seek agreement over disagreement. We mitigated these risks by gathering a diverse group of experts, encouraging and reinforcing the autonomy of roles and positions (e.g., engineers, developers, human-centered design experts, and end-users), encouraging dissent (e.g., asking team members to critique process and decisions in ways that are constructive), and taking turns when presenting counter arguments. While we made significant progress in improving on the device, there remain important concerns to address in future iterations, such as enabling greater ease in donning and doffing with one hand and greater comfort wearing underneath typical clothing, which were identified as important features driving adoption. Another limitation is that we currently do not know if the human-centered approach used in the study actually improved clinical adoption, as the new device is not in the market. Further, the clinical effectiveness of the device is yet to be tested for post-stroke rehabilitation (currently in progress), although anecdotal experience from clinicians and emerging research from other patient populations indicate that the NewGait device effectively addresses gait and balance issues [26–28].

## Conclusions

In summary, we developed a low-cost, stroke-specific, gait and balance rehabilitation system using a novel human-centered design approach that involved empathy interviews and design sprints to collectively brainstorm ideas and incorporate end-user feedback in the device development process. We also performed a benchtop validation testing and biomechanical simulation testing to establish product durability and clinical feasibility and system usability evaluation to evaluate whether device usability was improved with the human-centered design. The findings of this study indicate that this iterative approach resulted in a stroke-specific NewGait device that met user needs effectively while offering enhanced durability, modularity, and usability compared to previous versions and competitive devices. Future research is needed to evaluate the short-term biomechanical adaptations and long-term clinical effectiveness of the NewGait device in a broad group of stroke population.

### Electronic supplementary material

Below is the link to the electronic supplementary material.

Supplementary Material 1

## Data Availability

No datasets were generated or analysed during the current study.

## Abbreviations

AFO: Ankle-foot orthoses
SUS: System Usability Scale
