# Supplementary material for: Human-centered design of a novel soft exosuit for post-stroke gait rehabilitation
Source: J Neuroeng Rehabil. 2024 Apr 24;21:62. doi: 10.1186/s12984-024-01356-3 (PMC11040835; doi:10.1186/s12984-024-01356-3)
Supplement: Supplementary file 1 — Supplementary Material 1 [file 12984_2024_1356_MOESM1_ESM.docx]

Supplemental Materials


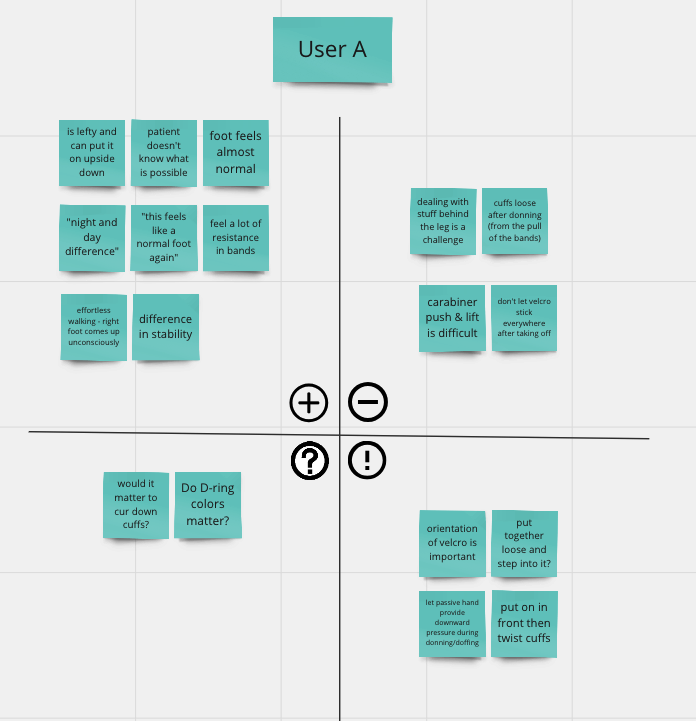


Supplemental Figure 1. Feedback from User A of design sprint #2


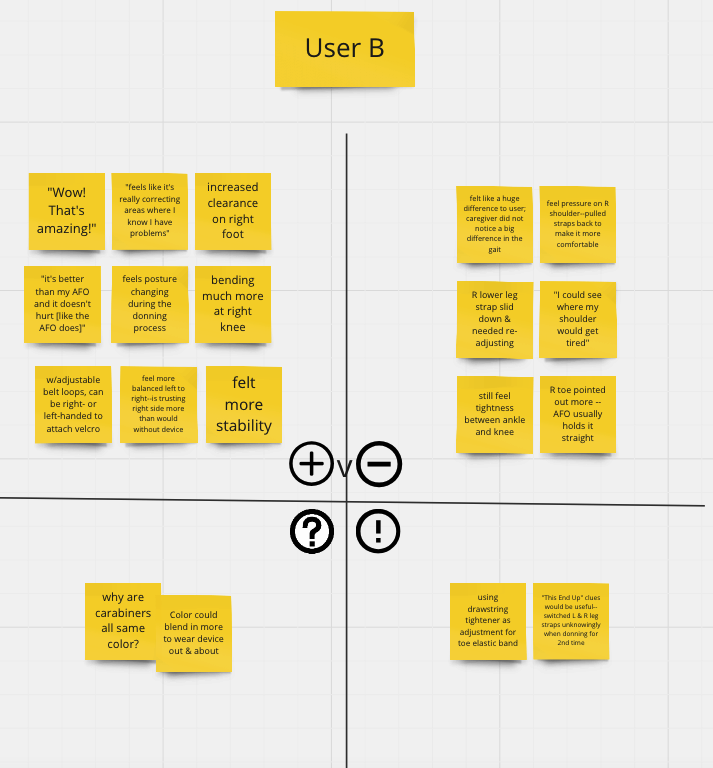


Supplemental Figure 2. Feedback from User B of design sprint #2


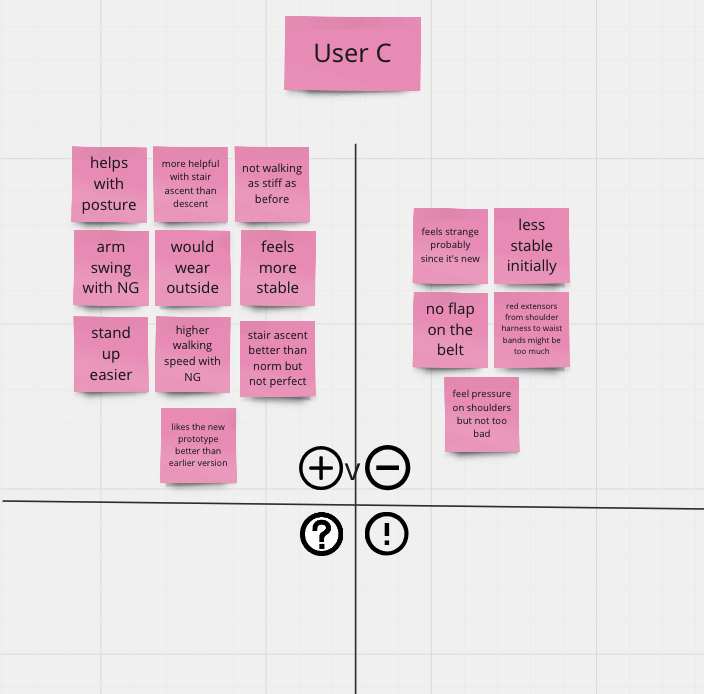


Supplemental Figure 3. Feedback from User C of design sprint #2


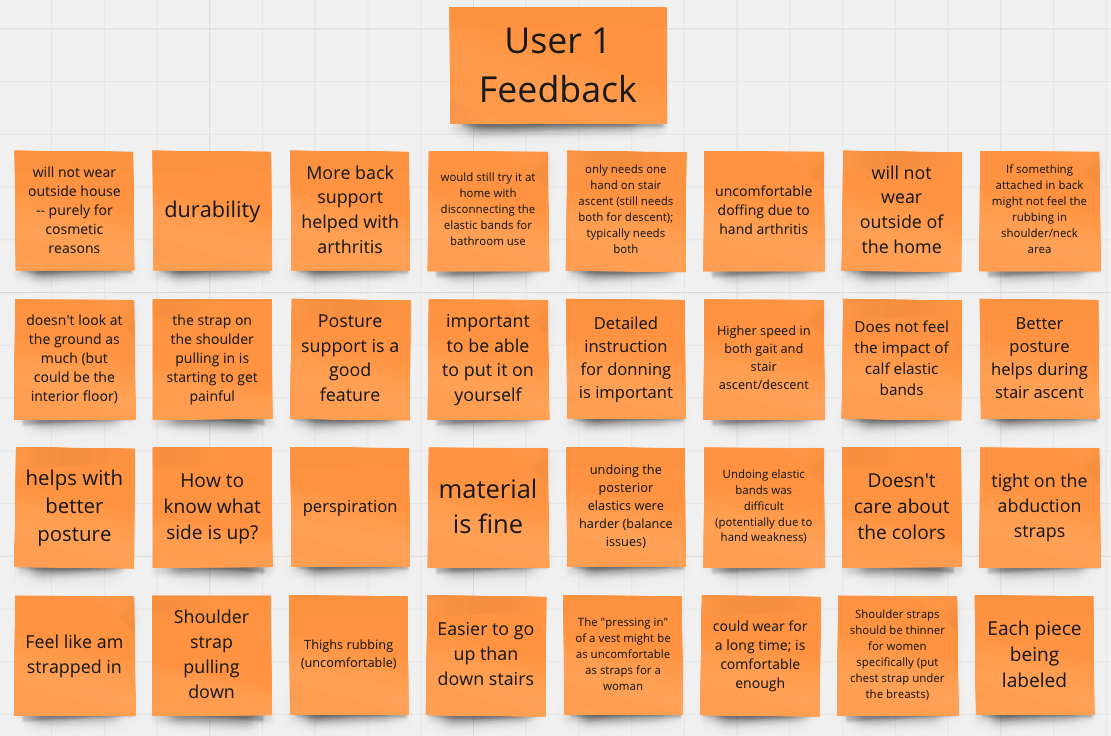


Supplemental Figure 4. Feedback from User 1 of design sprint #1


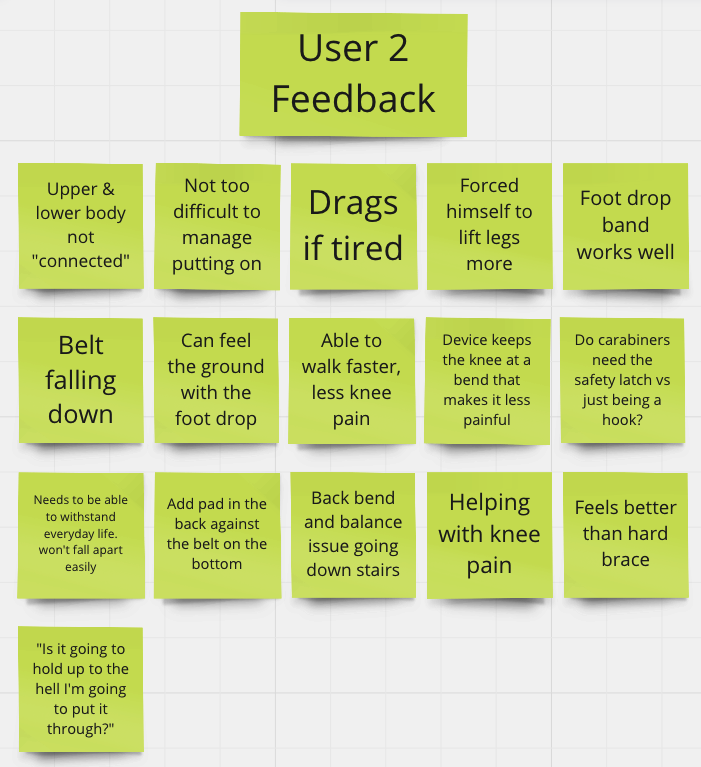


Supplemental Figure 5. Feedback from User 2 of design sprint #1


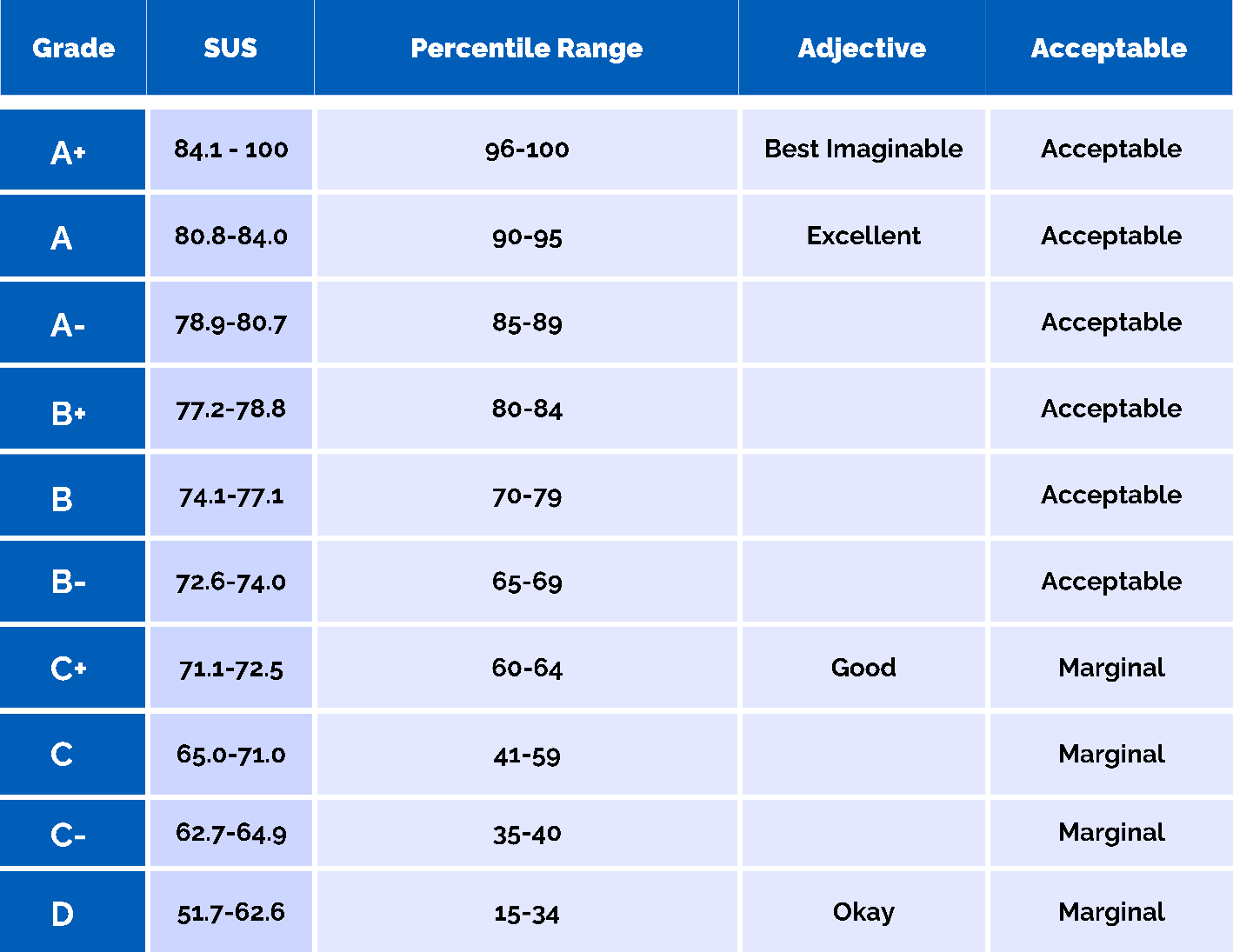


Supplemental Figure 6. Percentiles, grades, and adjectives to describe raw SUS. (adapted from Sauro, J) 5 Ways in Interpret a SUS Score: Measuring U; 2018 [Available from: https://measuringu.com/interpret-sus-score/


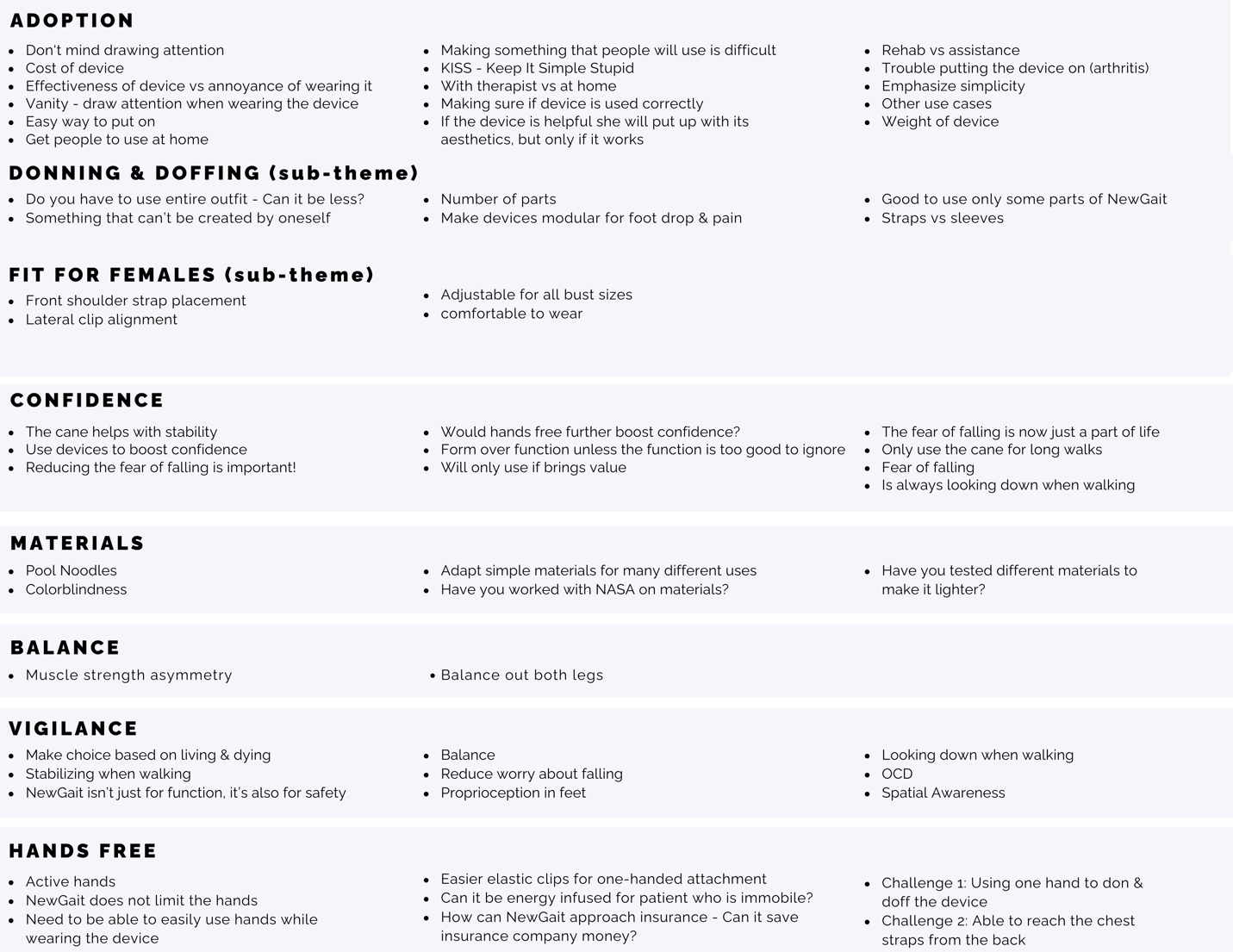


Supplemental Figure 7. A schematic of affinity mapping themes


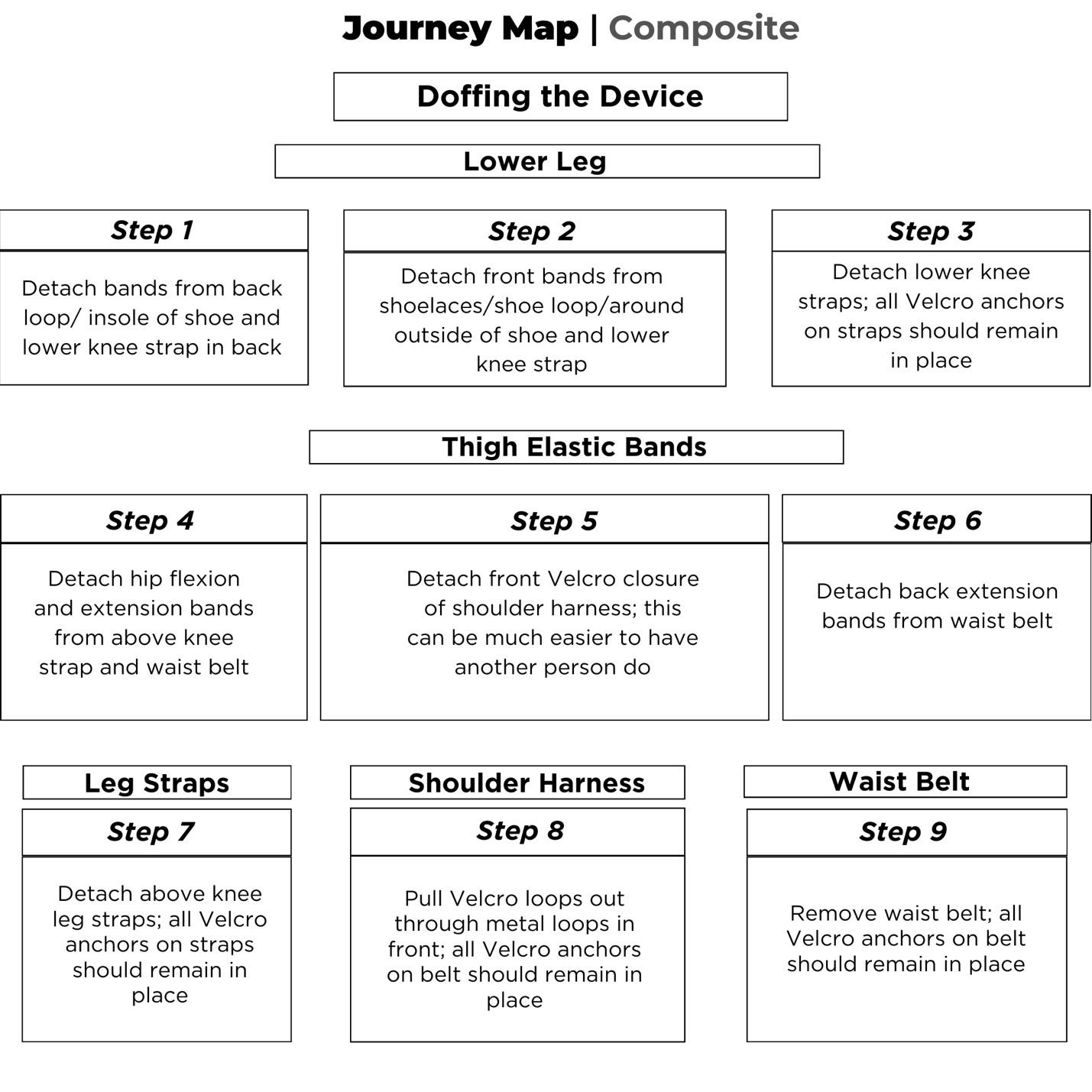


Supplemental Figure 8. A schematic of the device doffing journey map from design sprint #1 and #2


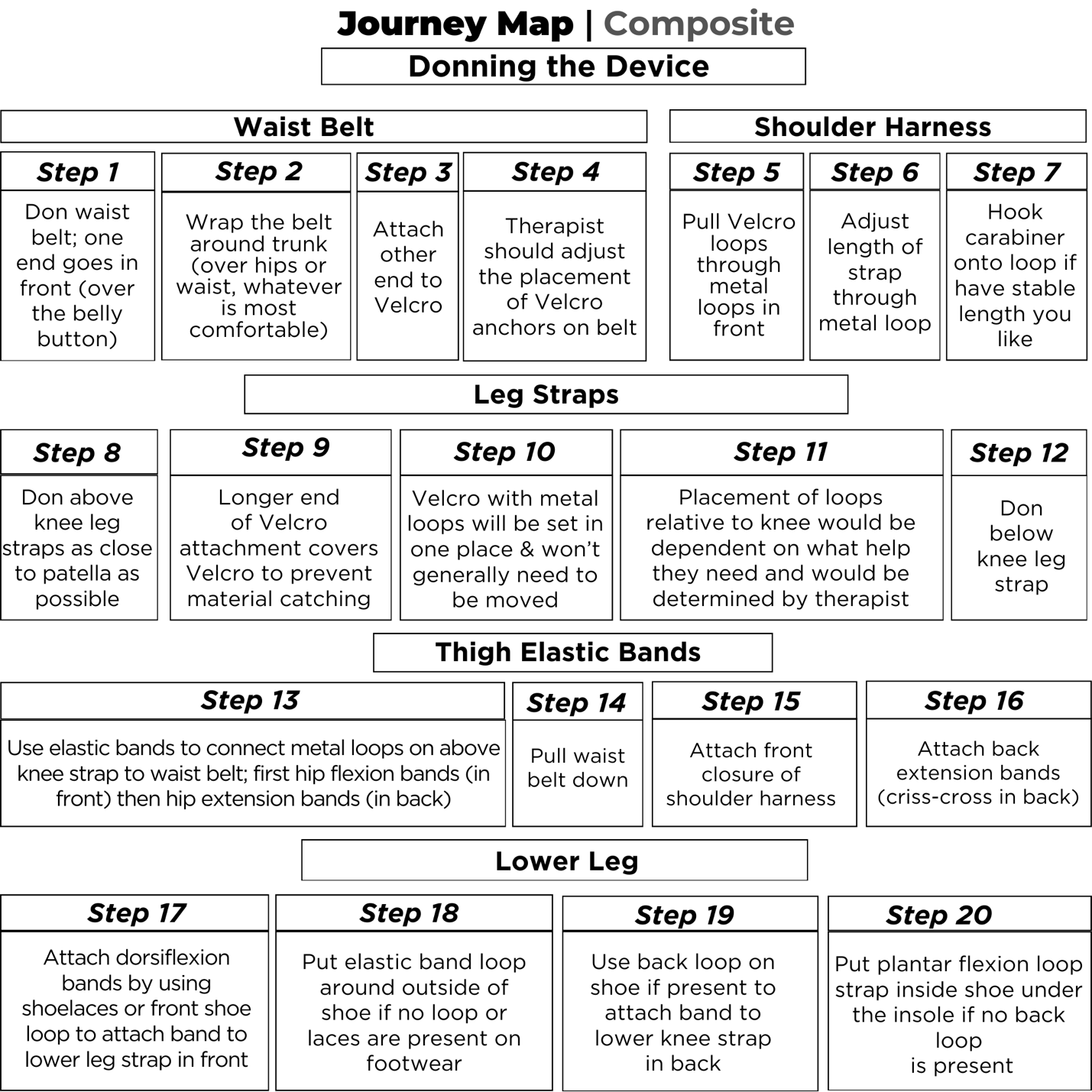


Supplemental Figure 9. A schematic of the device donning journey map from design sprint #1 and #2
